# Supplementary material for: Articulation points in complex networks
Source: Nat Commun. 2017 Jan 31;8:14223. doi: 10.1038/ncomms14223 (PMC5290321; doi:10.1038/ncomms14223)
Supplement: Supplementary Information — Supplementary Figures, Supplementary Tables, Supplementary Notes and Supplementary References. [file ncomms14223-s1.pdf]

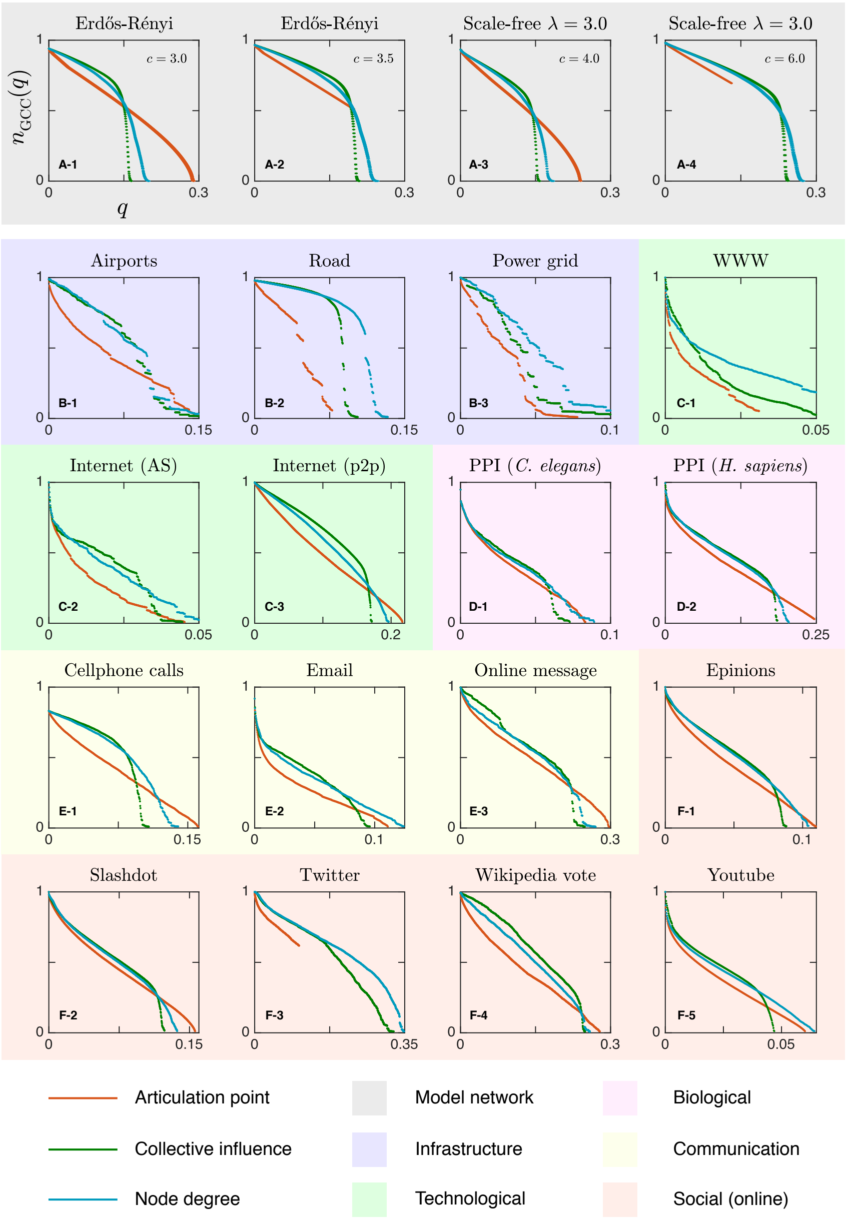

Supplementary Figure 1. Comparison of different network destruction strategies in reducing the size of the giant connected component for both model networks (**A**) and real-world networks (**B-F**). For model networks, the data points and error bars (defined as s.e.m.) are determined from 32 independent network instances of size $N = 10^5$. The real-world networks used in this figure are: openflights (**B-1**), RoadNet-TX (**B-2**), PG-WestState (**B-3**), nd.edu (**C-1**), oregon2-010331 (**C-2**), p2p-Gnutella31 (**C-3**), *Caenorhabditis elegans* (**D-1**), *Homo sapiens* (**D-2**), Cellphone (**E-1**), Email-Enron (**E-2**), UCIonline (**E-3**), Epinions (**F-1**), Slashdot-1 (**F-2**), Twitter (**F-3**), WikiVote (**F-4**), Youtube (**F-5**) (see Supplementary Note 7 for details of the real-world networks).

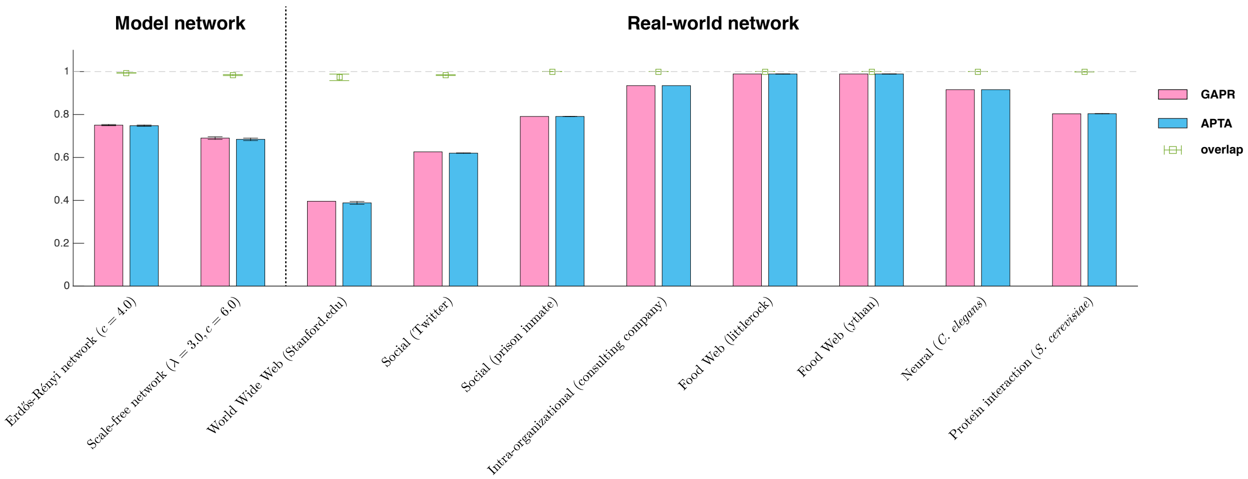

Supplementary Figure 2. The relative sizes of the two RGBs associated with the GAPR (pink bars) and the APTA (blue bars) processes, and the overlap between the two RGBs (green squares) in model networks and real-world networks. For each type of model network, the results (with error bars defined as s.e.m.) are obtained from 32 independent network instances with $N = 10^5$. For each real-world network, the results (with error bars defined as s.e.m.) are averaged over 32 independent realizations of APTA processes.

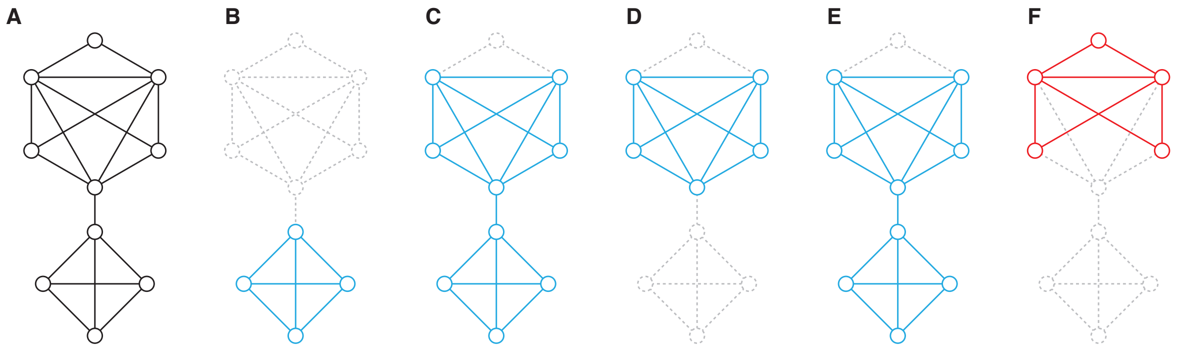

Supplementary Figure 3. Core-periphery structures. Different cores identified through various traditional decomposition methods in a small example graph (A) are shown (highlighted in blue): (B) the maximum clique; (C) the k-core with $k = 3$; (D) the edge-t-core with $t = 2$; (D) the vertex-t-core with $t = 3$. The RGB (highlighted in red) of the same graph is illustrated in (F).

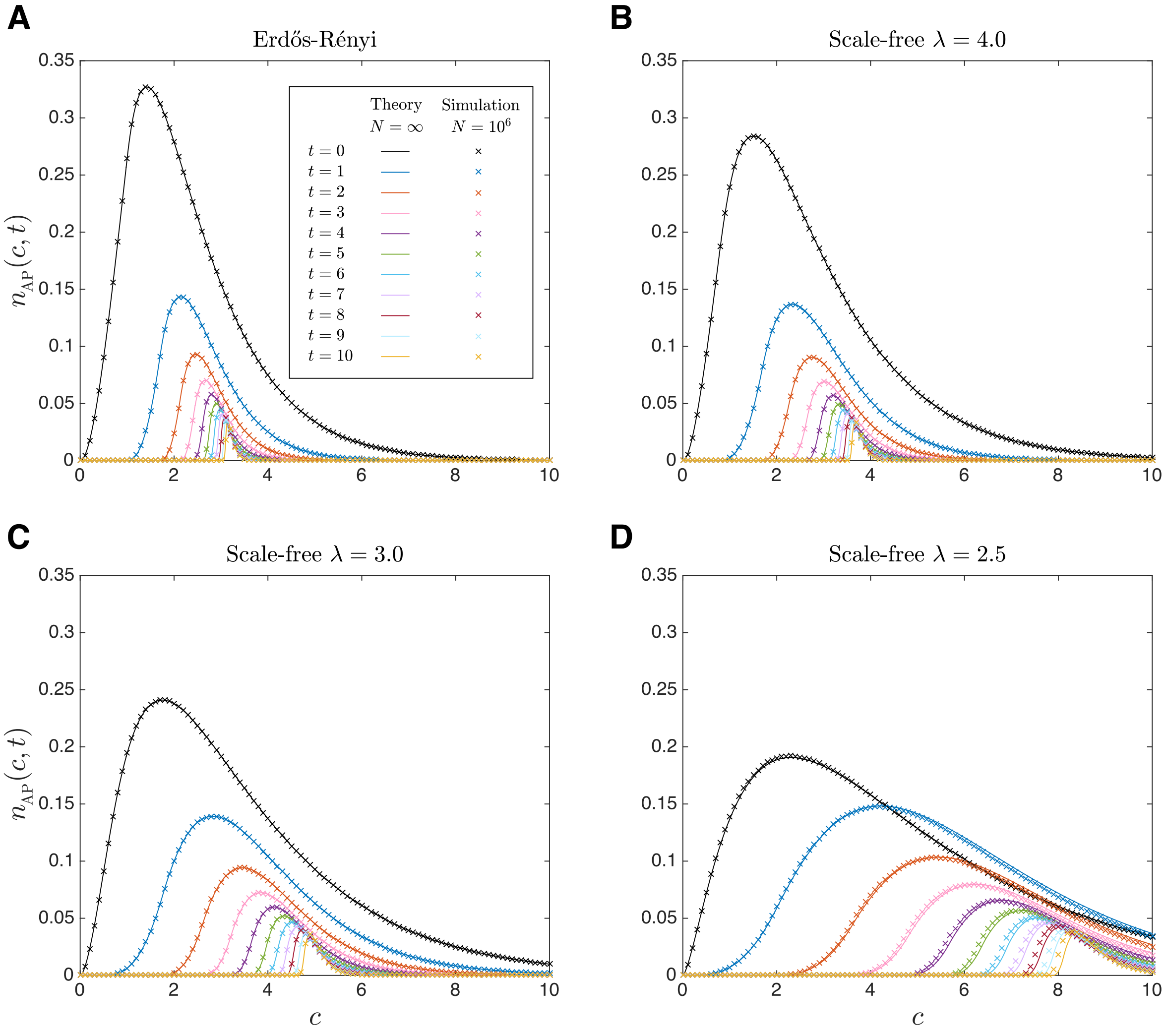

Supplementary Figure 4. The fraction of APs, $n_{\mathrm{AP}}(c,t)$, as a function of mean degree c at different time steps t in the GAPR process, for ER networks (A) and SF networks with different degree exponents $\lambda = 4.0, 3.0, 2.5$ (B-D). Symbols are numerical results calculated from the GAPR procedure on finite networks constructed with ER model (A) and the static model (B-D) with $N = 10^6$. The data points and error bars (defined as s.e.m.) are determined from 128 independent network realizations. Lines are analytical results for infinitely large system ($N \to \infty$).

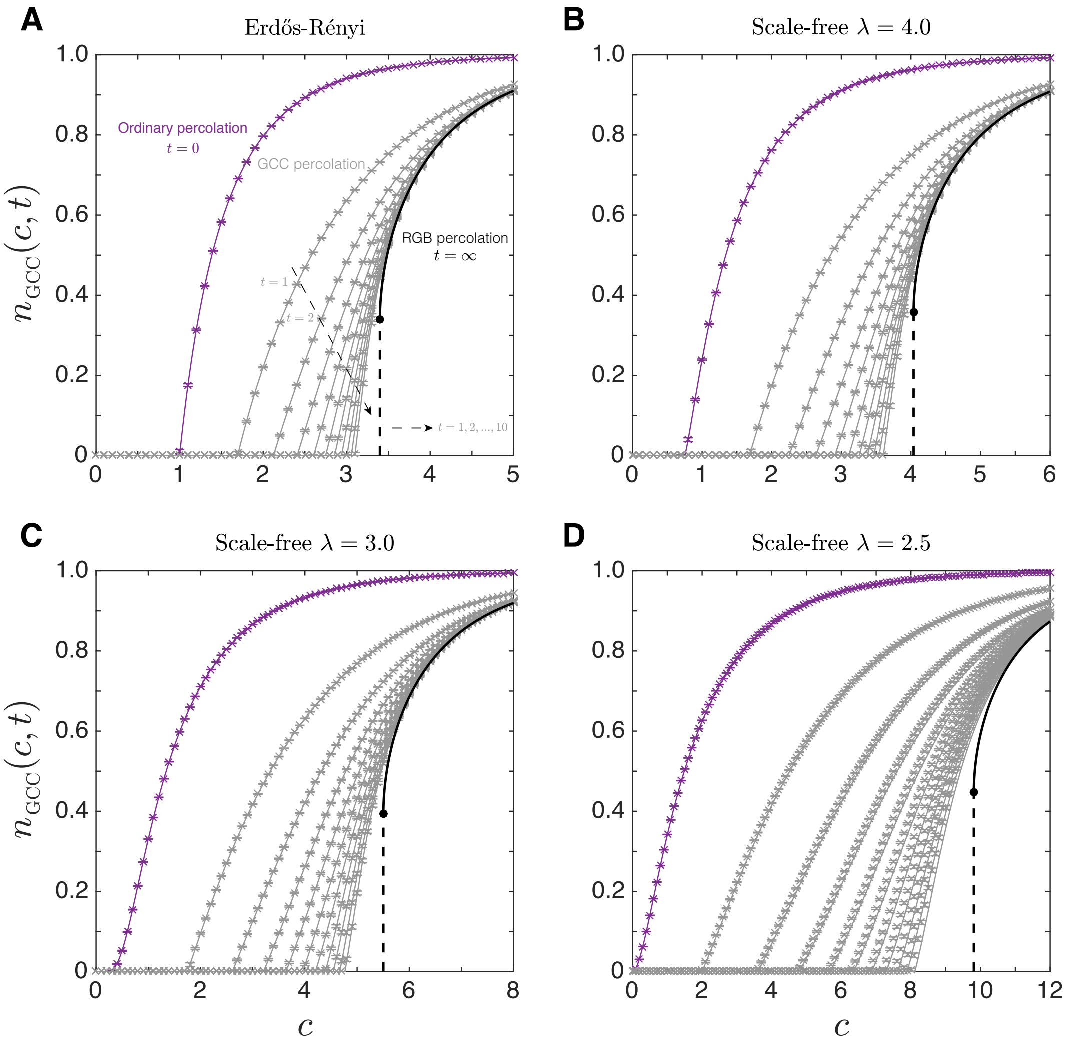

Supplementary Figure 5. The relative size of the GCC, $n_{\mathrm{GCC}}(c,t)$, as a function of mean degree c at different time steps t in the GAPR process, for ER networks (A) and SF networks with different degree exponents $\lambda = 4.0, 3.0, 2.5$ (B-D). Symbols are numerical results calculated by performing a given steps of GAPR procedure on finite networks constructed with ER model (A) and the static model (B-D) with $N = 10^6$. The data points and error bars (defined as s.e.m.) are determined from 128 independent network realizations. Thin lines are analytical results for $n_{\mathrm{GCC}}(c,t)$ with finite t, and thick lines are analytical results for the relative size of the RGB, which is $n_{\mathrm{GCC}}(c,\infty)$.

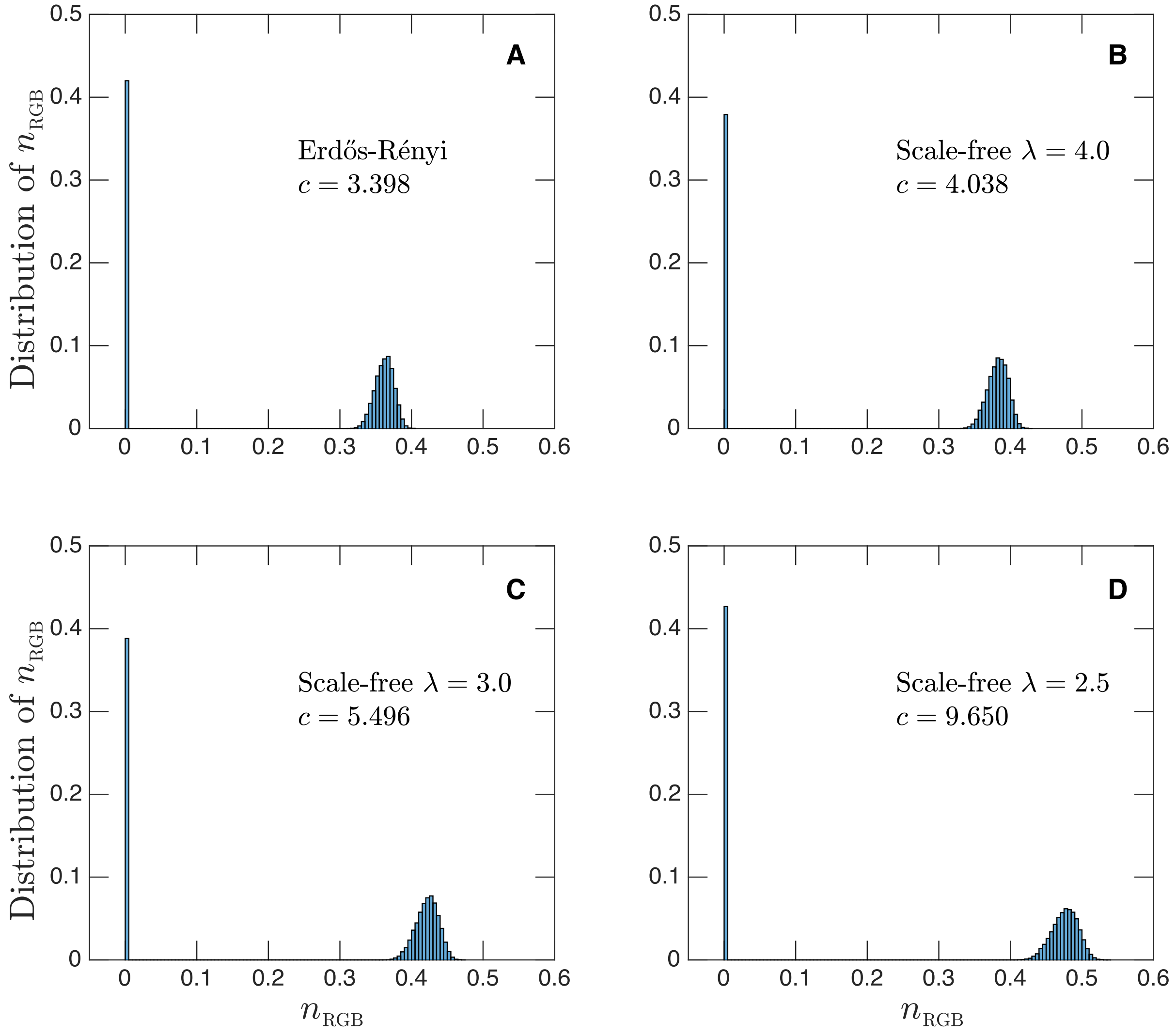

Supplementary Figure 6. The distribution of the relative size of the RGB, n_{RGB}, at criticality for both ER network (A) and SF networks with different exponents $\lambda = 4.0, 3.0, 2.5$ (B-D). Each distribution is generated by performing GAPR process on 12,800 different finite networks of size $N = 10^6$.

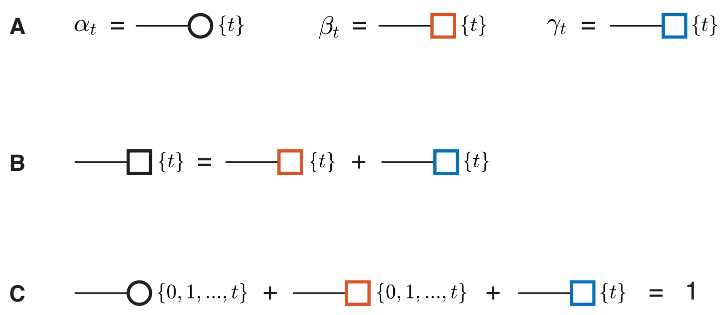

Supplementary Figure 7. (A) Diagrammatic notations for the probabilities that, following a randomly chosen link to one of its end nodes, we arrive at α_t-node, β_t-node, and γ_t-node, respectively, at time step t. (B) The probability that an end node of a randomly chosen link belongs to the GCC at time step t is given by $\beta_t + \gamma_t$. (C) Diagrammatic representation of the normalization condition of the three sets of probabilities $\{\alpha_0, \alpha_1, ...\}$, $\{\beta_0, \beta_1, ...\}$, and $\{\gamma_0, \gamma_1, ...\}$.

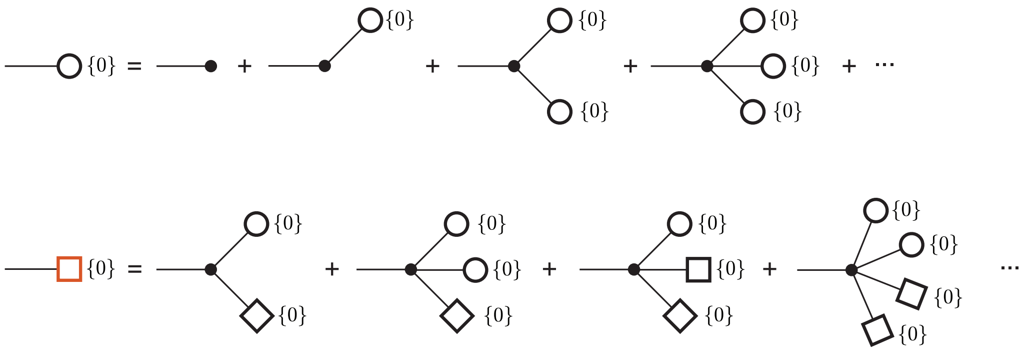

Supplementary Figure 8. Diagrammatic representations of the self-consistent equations for α_0 and β_0.

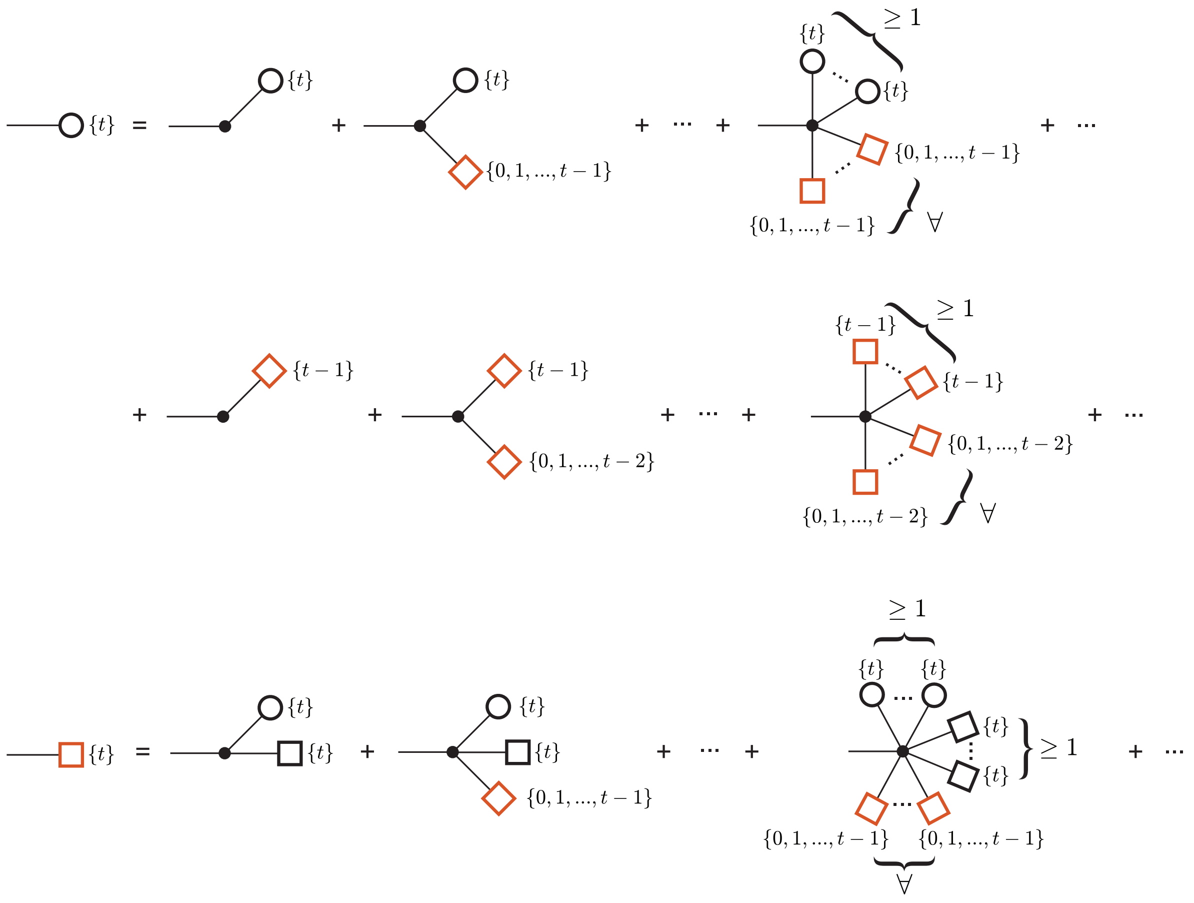

Supplementary Figure 9. Diagrammatic representations of the self-consistent equations for α_t and β_t.

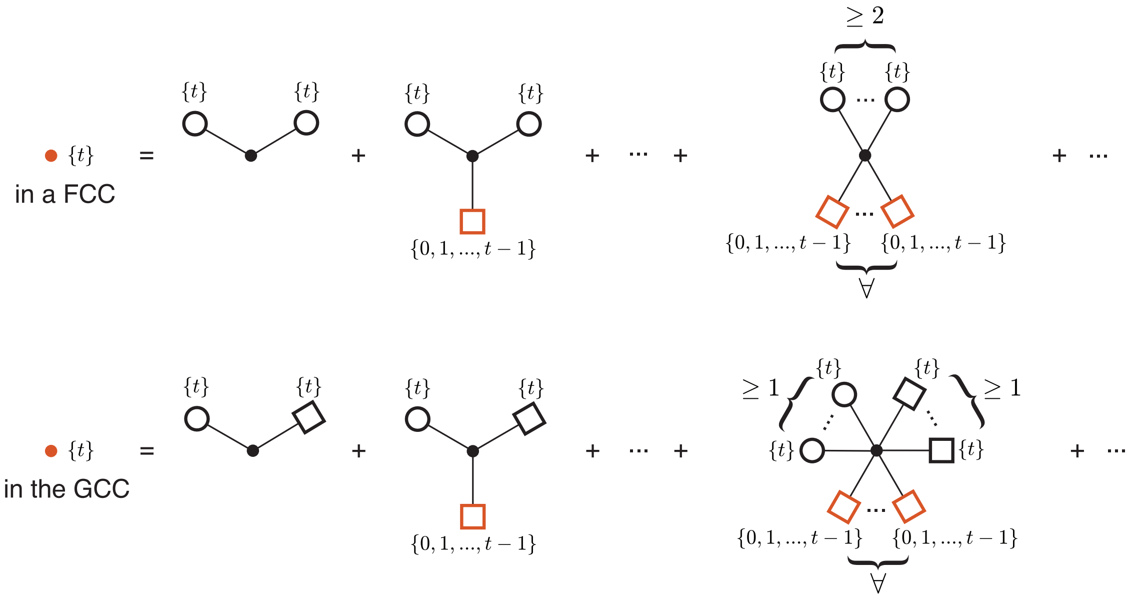

Supplementary Figure 10. Diagrammatic representations of fraction of APs in FCCs, $n_{\rm AP}^{\rm FCC}(t,c)$, and the GCC, $n_{\rm AP}^{\rm GCC}(t,c)$ (Eqs. 14 and 15).

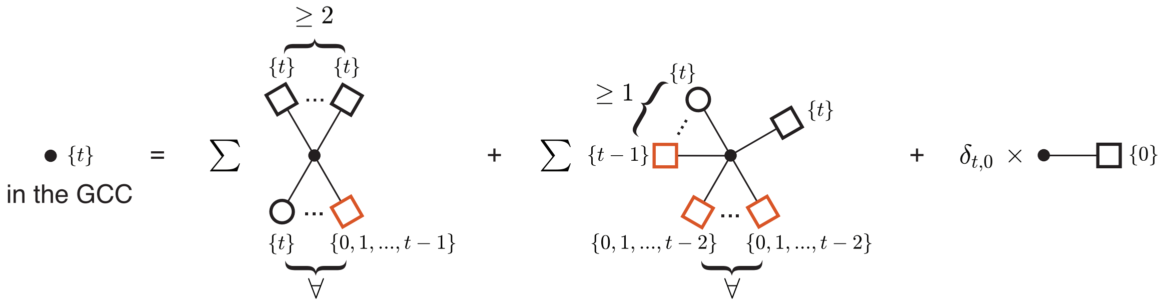

Supplementary Figure 11. Diagrammatic representation of relative size of the GCC, $n_{\mathrm{GCC}}(t,c)$, after performing t steps of GAPR (Eq. 19).

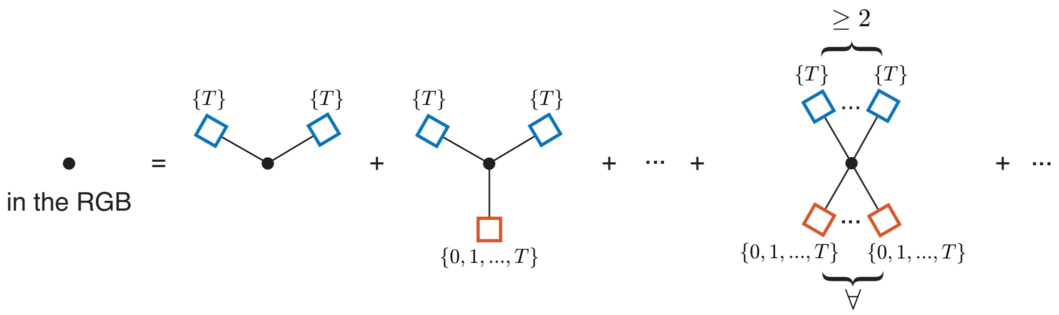

Supplementary Figure 12. Diagrammatic representation of relative size of the RGB, $n_{\mathrm{RGB}}(c)$ (Eq. 22).

Supplementary Table 1. **Air traffic networks**. For each network, we show its name and reference; number of nodes (N) and edges (L); and a brief description.

Name	N	L	description
USairport500 [1]	500	5,960	Top 500 busiest commercial airports in US
USairport-2010 [2]	1,574	28,236	US airport network in 2010
openflights [2]	2,939	30,501	non-US-based airport network

Supplementary Table 2. **Road networks**. For each network, we show its name and reference; number of nodes (N) and edges (L); and a brief description.

Name	N	L	description
RoadNet-CA [3]	1,965,206	5,533,214	California road network
RoadNet-PA [3]	1,088,092	3,083,796	Pennsylvania road network
RoadNet-TX [3]	1,379,917	3,843,320	Texas road network

Supplementary Table 3. **Power grids (PG)**. For each network, we show its name and reference; number of nodes (N) and edges (L); and a brief description.

Name	N	L	description
PG-Texas [4]	4,889	5,855	Power grid in Texas
PG-WestState [5]	4,941	6,594	High-voltage power grid in the Western States in US

Supplementary Table 4. **Internet: Autonomous Systems (AS)** [3]. For each network, we show its name; number of nodes (N) and edges (L); and a brief description.

Name	N	L	description
as20000102	6,474	13,895	AS graph from January 02 2000
oregon1-010331	10,670	22,003	AS peering information inferred from Oregon route-views (I)
oregon1-010407	10,729	22,000	Same as above (at different time)
oregon1-010414	10,790	22,470	Same as above (at different time)
oregon1-010421	10,859	22,748	Same as above (at different time)
oregon1-010428	10,886	22,494	Same as above (at different time)
oregon1-010505	10,943	22,608	Same as above (at different time)
oregon1-010512	11,011	22,678	Same as above (at different time)
oregon1-010519	11,051	22,725	Same as above (at different time)
oregon1-010526	11,174	23,410	Same as above (at different time)
oregon2-010331	10,900	31,181	AS peering information inferred from Oregon route-views (II)
oregon2-010407	10,981	30,856	Same as above (at different time)
oregon2-010414	11,019	31,762	Same as above (at different time)
oregon2-010421	11,080	31,539	Same as above (at different time)
oregon2-010428	11,113	31,435	Same as above (at different time)
oregon2-010505	11,157	30,944	Same as above (at different time)
oregon2-010512	11,260	31,304	Same as above (at different time)
oregon2-010519	11,375	32,288	Same as above (at different time)
oregon2-010526	11,461	32,731	Same as above (at different time)

Supplementary Table 5. **Internet: peer-to-peer (p2p) file sharing networks** [3]. For each network, we show its name; number of nodes (N) and edges (L); and a brief description.

Name	N	L	description
p2p-Gnutella04	10,876	39,994	Gnutella p2p file sharing network
p2p-Gnutella05	8,846	31,839	Same as above (at different time)
p2p-Gnutella06	8,717	31,525	Same as above (at different time)
p2p-Gnutella08	6,301	20,777	Same as above (at different time)
p2p-Gnutella09	8,114	26,013	Same as above (at different time)
p2p-Gnutella24	26,518	65,369	Same as above (at different time)
p2p-Gnutella25	22,687	54,705	Same as above (at different time)
p2p-Gnutella30	36,682	88,328	Same as above (at different time)
p2p-Gnutella31	62,586	147,892	Same as above (at different time)

Supplementary Table 6. **Electronic circuits** [6]. For each network, we show its name; number of nodes (N) and edges (L); and a brief description.

Name	N	L	description
s838	512	819	Electronic sequential logic circuit
s420	252	399	Same as above
s208	122	189	Same as above

Supplementary Table 7. **World Wide Web (WWW)**. For each network, we show its name and reference; number of nodes (N) and edges (L); and a brief description.

Name	N	L	description
stanford.edu [3]	281,903	1,992,636	WWW from nd.edu domain
nd.edu [7]	325,729	1,090,108	WWW from stanford.edu domain

Supplementary Table 8. **Food webs (FW)**. For each network, we show its name and reference; number of nodes (N) and edges (L); and a brief description.

Name	N	L	description
baydry [8]	128	2,137	FW at Florida Bay, Dry Season
baywet [8]	128	2,106	FW at Florida Bay, Wet Season
Chesapeake [9]	39	177	FW at Chesapeake Bay Mesohaline Net
ChesLower [10]	37	178	FW at Lower Chesapeake Bay in Summer
ChesMiddle [10]	37	209	FW at Middle Chesapeake Bay in Summer
ChesUpper [10]	37	215	FW at Upper Chesapeake Bay in Summer
CrystalC [11]	24	125	FW at Crystal River Creek (Control)
CrystalD [11]	24	100	FW at Crystal River Creek (Delta Temp)
cypdry [12]	71	640	FW at Cypress, Dry Season
cypwet [12]	71	631	FW at Cypress, Wet Season
Everglades [13]	69	916	FW at Everglades Graminoid Marshes
Florida [8]	128	2,106	FW at Florida Bay Trophic Exchange Matrix
gramdry [13]	69	915	FW at Everglades Graminoids, Dry Season
gramwet [13]	69	916	FW at Everglades Graminoids, Wet Season
grassland [14]	88	137	FW at Grassland
littlerock [15]	183	2,494	FW at Little Rock lake.
mangdry [16]	97	1,491	FW at Mangrove Estuary, Dry Season
mangwet [16]	97	1,492	FW at Mangrove Estuary, Wet Season
Maspalomas [17]	24	82	FW at Charca de Maspalomas
Michigan [12]	39	221	FW at Lake Michigan Control network
Mondego [16]	46	400	FW at Mondego Estuary - Zostrea site
Narragan [16]	35	220	FW at Narragansett Bay Model
Rhode [12]	20	53	FW at Rhode River Watershed - Water Budget
seagrass [18]	49	226	FW at St. Marks Seagrass.
silwood [19]	154	370	FW at Silwood Park
StMarks [20]	54	356	FW at St. Marks River (Florida) Flow network
stmartin [21]	45	224	FW at St. Martin Island
ythan [14]	135	601	FW at Ythan Estuary

Supplementary Table 9. **Neural network**. For the network, we show its name and reference; number of nodes (N) and edges (L); and a brief description.

Name	N	L	description
C.elegans [5]	297	2,148	Neural network of *C.elegans*

Supplementary Table 10. **Transcriptional regulatory networks (TRN)**. For each network, we show its name and reference; number of nodes (N) and edges (L); and a brief description.

Name	N	L	description
TRN-Yeast-Babu [22]	4,441	12,864	Transcriptional regulatory network of *S.cerevisiae*
TRN-Yeast-Alon [6]	688	1,078	Same as above (compiled by different group)
TRN-EC-RDB64 [23]	1,550	3,234	Transcriptional regulatory networkof *E.coli*
TRN-EC-Alon [6]	418	519	Same as above (compiled by different group)

Supplementary Table 11. **Protein-protein interaction (PPI) networks of different organisms** [24]. For each network, we show its name; and number of nodes (N) and edges (L).

Name	N	L
Arabidopsis thaliana (Columbia)	8,149	26,578
Bos taurus	394	405
Caenorhabditis elegans	3,941	8,641
Candida albicans (SC5314)	370	412
Danio rerio	235	281
Drosophila melanogaster	8,212	47,730
Emericella nidulans (FGSC A4)	64	71
Escherichia coli (K12/MG1655)	136	127
Gallus gallus	336	409
Hepatitus C Virus	112	164
Homo sapiens	18,632	224,270
Human Herpesvirus 1	139	183
Human Herpesvirus 4	223	295
Human Herpesvirus 5	71	79
Human Herpesvirus 8	140	191
Human Immunodeficiency Virus 1	1,028	1,786
Mus musculus	8,512	22,623
Oryctolagus cuniculus	182	198
Plasmodium falciparum (3D7)	1,227	2,545
Rattus norvegicus	3,313	5,357
Saccharomyces cerevisiae (S288c)	6,481	333,833
Schizosaccharomyces pombe (972h)	3,963	67,796
Solanum lycopersicum	25	86
Sus scrofa	87	75
Xenopus laevis	479	683

Supplementary Table 12. **Communication networks**. For each network, we show its name and reference; number of nodes (N) and edges (L); and a brief description.

Name	N	L	description
Cellphone [25]	36,595	91,826	Call network of cell phone users
Email-Enron [3]	36,692	367,662	Email communication network from Enron
Email-EuAll [3]	265,214	420,045	Email network from a large European research institute
Email-epoch [26]	3,188	39,256	Email network in a university
UCIonline [27]	1,899	20,296	Online message network of students at UC, Irvine
WikiTalk [3]	2,394,385	5,021,410	Wikipedia talk network

Supplementary Table 13. **Social networks**. For each network, we show its name and reference; number of nodes (N) and edges (L); and a brief description.

Name	N	L	description
Epinions [28]	75,888	508,837	Who-trusts-whom network of Epinions.com
college student [29, 30]	32	96	Social networks of positive sentiment (college students)
prison inmate [29, 30]	67	182	Same as above (prison inmates)
Slashdot-1 [3]	77,357	516,575	Slashdot social network
Slashdot-2 [3]	81,871	545,671	Same as above (at different time)
Slashdot-3 [3]	82,144	549,202	Same as above (at different time)
Slashdot-4 [3]	77,360	905,468	Same as above (at different time)
Slashdot-5 [3]	82,168	948,464	Same as above (at different time)
Twitter [3]	81,306	2,420,766	Social circles from Twitter
WikiVote [3]	7,115	103,689	Wikipedia who-votes-on-whom network
Youtube [3]	1,134,890	2,987,624	Youtube online social network

Supplementary Table 14. **Intra-organizational networks**. For each network, we show its name and reference; number of nodes (N) and edges (L); and a brief description.

Name	N	L	description
Freemans-1 [31]	34	695	Social network of network researchers
Freemans-2 [31]	34	830	Same as above (at different time)
Freemans-3 [31]	32	460	Same as above (at different time)
Consulting-1 [32]	46	879	Social network from a consulting company
Consulting-2 [32]	46	858	Same as above (different evaluation)
Manufacturing-1 [32]	77	2,326	Social network from a manufacturing company
Manufacturing-2 [32]	77	2,228	Same as above (different evaluation)

Supplementary Note 1 - Articulation-point-targeted attack (APTA)

A common measure of the structural integrity of a network is the size of its giant connected component (GCC). Articulation points (APs) are natural targets if we aim for immediate damage to the GCC. To quickly destruct the GCC, we can target the most destructive AP whose removal will cause the most nodes disconnected from the GCC. This leads to a brute-force strategy of network destruction:

Step-1: Identify all the APs in the current network, which can be achieved by a linear-time algorithm based on depth-first search [33].

Step-2: For each AP, calculate its "destructivity", i.e. how many nodes will be disconnected from the GCC after its removal.

Step-3: Rank all the APs based on their destructivities, and remove the most destructive one together with the links attaching to it. (In case the most destructive APs are not unique, randomly choose one of them to remove.)

Step-4: Repeat steps 1-3 until the network is totally destroyed or left with a residual giant biconnected component (RGB), in which no APs exist.

Hereafter we call this iterative process AP-targeted attack.

To demonstrate the efficiency of APTA in reducing the size of the GCC, we compare it with two other network destruction strategies based on the following node centrality measures:

- **Degree**. In this strategy, we iteratively remove the node with the highest degree in the current network [34, 35]. (In case the highest-degree nodes are not unique, we randomly choose one of them to remove.) Degrees of the remaining nodes will be recalculated after each node removal.

- **Collective influence**. The collective influence (CI) of node i is defined as the product of its reduced degree $(k_i - 1)$ and the total reduced degree of all nodes at distance l from it, i.e. $\mathrm{CI}_l(i) = (k_i - 1)\sum_{j\in\partial\mathrm{Ball}(i,l)}(k_j - 1)$, where $\partial\mathrm{Ball}(i,l)$ is the frontier of a ball of radius l centered at node i (i.e. the set of the l-th nearest neighbors of node i) [36]. In each step, we compute the CI of every node, and remove the node

with the largest CI. (In case the highest-CI nodes are not unique, we randomly choose one of them to remove.) It has been shown that, in locally tree-like large networks, the CI-based attack gives the optimal threshold, i.e. the minimal fraction of removed nodes that totally destroys the GCC [36]. Note that, for $l = 0$, this method is reduced to the degree-based network destruction.

We compute the relative size of the GCC, denoted as $n_{\rm GCC}$, as a function of the fraction of removed nodes q by using the three above-mentioned network destruction strategies in two classical configuration models (Supplementary Fig. 1A) and five different types of real-world networks (including technological, infrastructure, biological, communication, and online social networks) (Supplementary Figs. 1B-F). Interestingly, we find that, given a limited "budget" (i.e. a small fraction of nodes to be removed), APTA is the most efficient strategy in reducing GCC for both model and real networks (Supplementary Fig. 1). It turns out that even though AP removal maximizes only the *local* damage to the network in every step, and does not aim for a systemic or global breakdown of the network, for a certain range of q this is highly efficient in damaging the entire system. Moreover, in some of the networks, such as the road network (Supplementary Fig. 1B-2), the power grid (Supplementary Fig. 1B-3), and the World Wide Web (WWW) (Supplementary Fig. 1C-1), APTA is optimal even in the whole range of q. The reason that for those networks APTA leads to smaller critical q than the state-of-the-art CI-based method is because these networks are either spatially embedded or/and rich in loops, in which the CI-based method is not guaranteed to be efficient.

Note that, even for networks with rather big RGBs (Supplementary Figs. 1A-2, A-4, F-3), ATPA is still very efficient till the GCC becomes biconnected (or becomes an RGB), where the process of APTA terminates.

Supplementary Note 2 - Residual giant biconnected component

The RGB can also be obtained by the process of greedy APs removal (GAPR). Performing GAPR on a given network consists of the following steps:

Step-1: Identify all the APs in the current network by the depth-first search algorithm [33].

Step-2: Simultaneously remove all the APs and the links attaching to them in the current network.

Step-3: Repeat steps 1-2 until no APs exist in the network.

For a general network of finite size, GAPR and APTA do not necessarily yield exactly the same RGB. In fact, the RGB obtained from the former is unique (because the GAPR process is deterministic), while the RGB obtained from the latter could be history-dependent (there may be multiple most destructive APs in each step).

For a given network, we denote the sets of nodes in the two RGBs associated with the two processes as V_{GAPR} and V_{APTA}, respectively. The overlap between the two RGBs can be measured by the classical Jaccard index:

$$\text{overlap} = \frac{|V_{\mathrm{GAPR}} \bigcap V_{\mathrm{APTA}}|}{|V_{\mathrm{GAPR}} \bigcup V_{\mathrm{APTA}}|}. \tag{1}$$

In Supplementary Fig. 2, we show the relative sizes of the two RGBs, as well as the overlap between them. We find that the two RGBs significantly overlap for both model networks and real-world networks.

Supplementary Note 3 - Core-periphery structure

Core-periphery structures are commonly found in real-world complex networks [37–40]. They typically consist of a dense cohesive core and a sparse, loosely connected periphery. Revealing these core-periphery structures is very important to understand the structure and function of real-world complex networks. Traditional methods or algorithms used to identify the core of a network include maximum clique [41, 42], k-core decomposition [43, 44], t-core decomposition [40, 45, 46], and so on.

Here we briefly discuss the relationship between the cores identified through traditional methods and the RGB identified through our GAPR process:

- **Maximum clique**. In a undirected graph, a clique is a subset of vertices such that its induced subgraph is complete (any two distinct vertices in the clique are adjacent). There could be many cliques in a network, and the maximum clique is the one with the most nodes. It should be noted that identifying the maximum clique in a network is generally an NP-complete problem.

- ***k*-core**. In a undirected graph, a k-core is the largest subgraph that each vertex participates in at least k links within the subgraph. The k-core number of such subgraph is k.

- ***t*-core**. By definition, there are two types of t-core:

  - **edge-*t*-core** [45]. In a undirected graph, an edge-t-core is the largest subgraph that each **edge** is contained within at least t different triangles in the subgraph. The edge-t-core number of such subgraph is referred to as t.

  - **vertex-*t*-core** [46]. In a undirected graph, a vertex-t-core is the largest subgraph that each **vertex** is contained within at least t different triangles in the subgraph. The vertex-t-core number of such subgraph is referred to as t.

In Supplementary Fig. 3, we show the maximum clique, the k-core with $k = 3$, the edge-t-core with $t = 2$, the vertex-t-core with $t = 3$, as well as the RGB in a small graph. The RGB is topologically different from the various cores identified through traditional methods. Moreover, compared with other cores that typically consist of high-degree nodes, the RGB identified through GAPR may also include low-degree nodes, which can be functionally very

important but always ignored in traditional methods. Hence, our AP-based decomposition method provides us a new angle to analyze and understand the organization of real-world complex networks.

Supplementary Note 4 - Theoretical Framework

A. Local tree approximation

Local tree approximation assumes that in the thermodynamics limit (i.e. the network size $N \to \infty$) there are no finite loops in the network and only infinite loops exist. This approximation directly leads to three important network properties [35, 47–50]:

1. All of the finite connected components (FCCs) are trees. Loops (of infinite length) only exist in the GCC;
2. There can be only one GCC in the network [49];
3. For any node in the network, its neighbors become disconnected or independent from each other if the node and its attaching links are removed.

In our theoretical framework, we use the above local tree approximation to study the GAPR process in large uncorrelated random networks with arbitrary degree distribution $P(k)$ and finite mean degree $c = \sum_k kP(k)$. Note that the local tree approximation becomes exact for networks with finite second moment of the degree distribution, such as Erdős-Rényi (ER) networks or scale-free (SF) networks with degree exponent $\lambda > 3$. However, we can still use this approximation to study networks with diverging second moment of the degree distribution, such as SF networks with $\lambda \leq 3$. It has been demonstrated in literature that the local tree approximation obtains very accurate results for various network problems [49]. Actually, this approximation has been widely used in studying loopy networks [49, 51]. Here we find that it works very well for studying AP-related problems in SF networks with $\lambda \leq 3$ (see Supplementary Note 6 for numerical simulations).

B. Discrete-time dynamics of GAPR

The deterministic GAPR process is naturally associated with a temporal order. At $t = 0$, we have the original network. At $t = T$, i.e. the last time step of GAPR, no AP exists in the network, and the GAPR process terminates. At each time step t, we denote the fraction of APs and the relative size of the GCC as $n_{\mathrm{AP}}(c, t)$ and $n_{\mathrm{GCC}}(c, t)$, respectively, where c is

the mean degree of the original network. The RGB is nothing but the GCC at the last time step, whose relative size is denoted as $n_{\mathrm{RGB}}(c) = n_{\mathrm{GCC}}(c, T)$.

At each time step t during the GAPR process in a network $\mathcal{G}$, we classify the remaining nodes into the following three categories or states:

1. α_t-nodes: nodes that belong to FCCs at time t (note that by definition α_t-nodes include APs in FCCs);

2. β_t-nodes: nodes that are APs in the GCC at time t;

3. γ_t-nodes: nodes that are not APs and belong to the GCC at time t. Note that if a node is a γ_t-node at time t, this node must be γ_τ-node with $\tau < t$.

Note that the notations β_t and γ_t here have totally different meanings from the critical exponents mentioned in the main text.

According to the local tree approximation, the state of a randomly chosen node i can be determined by the states of its neighbors in $\mathcal{G}\backslash i$, i.e. the induced subgraph of $\mathcal{G}$ with node i and all its links removed. In order words, in order to determine the state of a node, we need to know the states of its neighbors. Therefore, at each time step t, we need to know the probability that, following a randomly chosen link to one of its end nodes, this node belongs to any of the above categories after this link is removed. These probabilities are denoted as α_t, β_t, and γ_t, respectively. Note that for convenience sake here we use the same notation to denote both the state of a node and the probability of a node in that state. To be precise, hereafter when we consider the state of a neighbor of a given node i, we mean the state of the neighbor in the induced subgraph $\mathcal{G}\backslash i$. Also, when we mention the state of an end node of a chosen link l, we mean the state of the node in $\mathcal{G}\backslash l$, i.e. the induced subgraph of $\mathcal{G}$ with link l removed. According to the definitions of β_t and γ_t, the probability that an end node of a randomly chosen link belongs to the GCC at time step t is just $\beta_t + \gamma_t$.

The GAPR process can be fully characterized by the three sets of probabilities $\{\alpha_0, \alpha_1, ...\}$, $\{\beta_0, \beta_1, ...\}$, and $\{\gamma_0, \gamma_1, ...\}$. Note that every node must belong to one of the three categories, which means the three sets of probabilities are not independent from each other. Specifically, at time step t, the probability γ_t can be derived by the other two sets of probabilities through the following normalization condition:

$$\sum_{\tau=0}^{t} \alpha_\tau + \sum_{\tau=0}^{t} \beta_\tau + \gamma_t = 1. \tag{2}$$

Supplementary Figure 7 shows the diagrammatic representations of these probabilities; the probability that an end node of a randomly chosen link belongs to the GCC at time step t; and the normalization equation 2.

Thanks to Eq. 2, hereafter all the equations will be written in terms of α_t and β_t only. We can calculate $\{\alpha_0, \alpha_1, ...\}$ and $\{\beta_0, \beta_1, ...\}$ in an iterative way. At first, we consider the initial time step $t = 0$. The self-consistent equations for α_0 and β_0 are given by

$$\alpha_0 = \sum_{k=1}^{\infty} Q(k) \left(\alpha_0\right)^{k-1} \tag{3}$$

$$\beta_0 = \sum_{k=3}^{\infty} Q(k) \left[1 - \left(1-\alpha_0\right)^{k-1} - \left(\alpha_0\right)^{k-1}\right], \tag{4}$$

where

$$Q(k) = kP(k)/c \tag{5}$$

is the degree distribution of the nodes that we arrive at by following a randomly chosen link (a.k.a. the excess degree distribution) [52, 53]. We derive the above equations based on the following observations:

- α_0-node: its neighbors can only be α_0-nodes;
- β_0-node: since it is an AP node, at least one of its neighbors is an α_0-node. Moreover, since it belongs to the GCC, at lease one of its neighbors is not an α_0-node.

Note that, due to the local tree approximation, removal of an AP in the GCC can only cause a finite number of nodes disconnected from the GCC, and there exists no such an AP that its removal breaks down the GCC into two infinitely large components. The diagrammatic representations of Eqs. 3 and 4 are shown in Supplementary Fig. 8.

For the t-th GAPR time step ($t > 0$), we can compute α_t and β_t as follows:

$$\alpha_t = \sum_{k=1}^{\infty} Q(k) \left(\alpha_t + \sum_{\tau=0}^{t-1} \beta_\tau\right)^{k-1} - \sum_{k=1}^{\infty} Q(k) \left(\sum_{\tau=0}^{t-2} \beta_\tau\right)^{k-1} \tag{6}$$

$$\begin{aligned}\beta_t =& \sum_{k=3}^{\infty} Q(k) \sum_{s=1}^{k-2} \binom{k-1}{s} \left(1 - \sum_{\tau=0}^{t} \alpha_\tau - \sum_{\tau=0}^{t-1} \beta_\tau\right)^{s} \\ & \times \sum_{r=1}^{k-1-s} \binom{k-1-s}{r} \left(\alpha_t\right)^r \left(\sum_{\tau=0}^{t-1} \beta_\tau\right)^{k-1-s-r} .\end{aligned} \tag{7}$$

The derivations of Eqs. 6 and 7 are based on the following observations:

- α_t-node: First of all, its neighbors can only be α_t-nodes or β_τ-nodes with $\tau < t$ (because if one of its neighbors is α_τ-node with $\tau < t$, this node will be an AP before time step t and hence would have already been removed; if one of its neighbor is β_t-node, this node will belong to the GCC at time step t). Second, its neighbors can not be simultaneously all β_τ-nodes with $\tau < t-1$. Otherwise this node will be a leaf node before time step $t-1$, and will become an isolated node before the t-th time step. In this case, we can not reach this node through a randomly chosen link at time step t.

- β_t-node: First of all, its neighbors can not be α_τ-nodes with $\tau < t$, otherwise this node would have been removed before time step t. Second, since it is an AP node, at least one of its neighbors is an α_t-node. Finally, since this node belongs to the GCC, at least one of its neighbors is neither α_t-node nor β_τ-node with $\tau < t$.

The diagrammatic representations of Eqs. 6 and 7 are shown in Supplementary Fig. 9.

Combining Eqs. 3, 4, 6, and 7, we obtain a complete set of discrete-time dynamic equations for the GAPR process:

$$\alpha_0 = G_1(\alpha_0) \tag{8}$$

$$\alpha_{t>0} = G_1\left(\alpha_t + \sum_{\tau=0}^{t-1}\beta_\tau\right) - G_1\left(\sum_{\tau=0}^{t-2}\beta_\tau\right) \tag{9}$$

$$\beta_t = G_1\left(1 - \sum_{\tau=0}^{t-1}\alpha_\tau\right) - G_1\left(1 - \sum_{\tau=0}^{t}\alpha_\tau\right) - G_1\left(\alpha_t + \sum_{\tau=0}^{t-1}\beta_\tau\right) + G_1\left(\sum_{\tau=0}^{t-1}\beta_\tau\right), \tag{10}$$

where

$$G_1(x) = \sum_k Q(k)x^{k-1} \tag{11}$$

is the generating function of the branching processes [48].

By solving the above self-consistent equations, we obtain $\{\alpha_0, \alpha_1, ...\}$ and $\{\beta_0, \beta_1, ...\}$, which govern the whole process of GAPR. With these two sets of probabilities, we can compute any quantities of interest, such as the total number of GAPR steps, the fraction of APs, the relative size of the GCC and the RGB, and so on.

C. Total number of GAPR steps

It should be noted from Eqs. 8, 9, and 10 that α_t and β_t depend not only on the previous time step $t-1$, but also on the entire history of GAPR, from the 0-th to the $(t-1)$-th

time steps. Hence to calculate T, the total number of GAPR steps, we have to solve the equations from the initial step to the T-th step when the GAPR process stops. Since the GAPR process terminates when there is no APs left in the network, T can be determined by requiring

$$\alpha_T = 0 \tag{12}$$

$$\beta_T = 0. \tag{13}$$

Note that, according to Eq. 9, β_{T-1} is also zero, otherwise α_T will have a non-zero solution.

D. Fraction of articulation points

Consider the fraction of APs at time step t during the GAPR process, $n_{\mathrm{AP}}(t,c)$, which also represents the probability that a randomly chosen node is an AP at t-th step. We can calculate $n_{\mathrm{AP}}(t,c)$ as the sum of the following two parts:

1. $n_{\mathrm{AP}}^{\mathrm{GCC}}(t,c)$: fraction of APs that belong to the GCC whose removal splits the GCC into a smaller GCC and many FCCs;

2. $n_{\mathrm{AP}}^{\mathrm{FCC}}(t,c)$: fraction of APs that belong to FCCs whose removal splits FCCs into smaller FCCs.

With similar theoretical considerations as deriving equations for α_t and β_t, we have

$$n_{\mathrm{AP}}^{\mathrm{FCC}}(t,c) = \sum_{k=2}^{\infty} P(k) \sum_{s=2}^{k} \binom{k}{s} (\alpha_t)^s \left(\sum_{\tau=0}^{t-1} \beta_\tau\right)^{k-s} \tag{14}$$

$$\begin{aligned} n_{\mathrm{AP}}^{\mathrm{GCC}}(t,c) =& \sum_{k=2}^{\infty} P(k) \sum_{s=1}^{k-1} \binom{k}{s} \left(1 - \sum_{\tau=0}^{t} \alpha_\tau - \sum_{\tau=0}^{t-1} \beta_\tau\right)^s \\ & \times \sum_{r=1}^{k-s} \binom{k-s}{r} \left(\sum_{\tau=0}^{t} \alpha_\tau\right)^r \left(\sum_{\tau=0}^{t-1} \beta_\tau\right)^{k-s-r} . \end{aligned} \tag{15}$$

These two equations can be understood as follows:

- AP in FCCs, $n_{\mathrm{AP}}^{\mathrm{FCC}}(t,c)$: First of all, since the node belongs to an FCC, its neighbor can only be α_t-nodes or β_τ-nodes with $\tau < t$. Second, since the node is an AP, at least two of its neighbors are α_t-nodes. Otherwise, it would be a leaf node or an isolated node at time step t.

- AP in the GCC, $n_{\rm AP}^{\rm GCC}(t,c)$: First of all, its neighbors can not be α_τ-nodes with $\tau < t$, otherwise this node would have already been removed before time step t. Second, since it is an AP node, at least one of its neighbors is an α_t-node. Finally, since this node belongs to the GCC, at least one of its neighbors is neither α_t-node nor β_τ-node with $\tau < t$.

The diagrammatic representations of Eqs. 14 and 15 are shown in Supplementary Fig. 10.

Combing Eqs. 14 and 15, we obtain the fraction of APs at time step t as

$$n_{\rm AP}(t,c) = n_{\rm AP}^{\rm FCC}(t,c)+n_{\rm AP}^{\rm GCC}(t,c) = G_0\left(1-\sum_{\tau=0}^{t-1}\alpha_\tau\right)-G_0\left(1-\sum_{\tau=0}^{t}\alpha_\tau\right)-c\alpha_t G_1\left(\sum_{\tau=0}^{t-1}\beta_\tau\right), \tag{16}$$

where

$$G_0(x) = \sum_k P(k)x^k \tag{17}$$

is the generating function of the degree distribution $P(k)$.

The fraction of APs in the original network, $n_{\rm AP}(c)$, can be obtained by substituting $t = 0$ into Eq. 16, yielding

$$n_{\rm AP}(c) = n_{\rm AP}(0,c) = 1 - G_0\left(1-\alpha_0\right) - c\alpha_0 G_1\left(0\right). \tag{18}$$

E. The giant connected component

Another quantity of interest is the relative size of the GCC, $n_{\rm GCC}(t,c)$, after performing a given t steps of GAPR in a network. We can compute $n_{\rm GCC}(t,c)$ as follows:

$$\begin{aligned} n_{\rm GCC}(t) =& \sum_{k=2}^{\infty} P(k)\sum_{s=2}^{k}\binom{k}{s}\left(\alpha_t+\sum_{\tau=0}^{t-1}\beta_\tau\right)^{k-s}\left(1-\sum_{\tau=0}^{t}\alpha_\tau-\sum_{\tau=0}^{t-1}\beta_\tau\right)^{s} \\ &+\sum_{k=2}^{\infty} P(k)\binom{k}{1}\left(1-\sum_{\tau=0}^{t}\alpha_\tau-\sum_{\tau=0}^{t-1}\beta_\tau\right)\sum_{r=1}^{k-1}\binom{k-1}{r}\left(\alpha_t+\beta_{t-1}\right)^{r}\left(\sum_{\tau=0}^{t-2}\beta_\tau\right)^{k-1-r} \\ &+\delta_{t,0}P(1)\left(1-\alpha_0\right), \end{aligned} \tag{19}$$

where the Kronecker delta function $\delta_{jk} = 1$ if $j = k$, and 0 otherwise. This equation can be understood as follows:

- For any node in the GCC at time step t, at least one of its neighbors belongs to the GCC at current time step. Moreover, none of its neighbors is α_τ-nodes with $\tau < t$, otherwise this node would have already been removed as an AP before time step t.

- The first term on the right hand side of Eq. 19 accounts for the nodes with at least two neighbors belonging to the GCC.
- The second term accounts for the nodes with only one neighbor in the GCC. It should be noted that, for each of these nodes, at least one of its neighbors is α_t-nodes or β_{t-1}-nodes. Otherwise, this node would be a leaf node before time step t, and thus will become an isolated node at time step t.
- We only consider nodes with degree larger than 2 in the first two terms. This is because any leaf nodes will become isolated ones right after the first step of GAPR. If we include the ordinary percolation as the 0-th time step in our GAPR process, leaf nodes can also belong to the GCC at the 0-th step, which is the third term.

The diagrammatic representation of Eq. 19 is shown in Supplementary Fig. 11. After some algebra, we can rewrite $n_{\mathrm{GGC}}(t,c)$ in terms of generating functions:

$$n_{\mathrm{GGC}}(t,c) = G_0\left(1-\sum_{\tau=0}^{t-1}\alpha_\tau\right) - G_0\left(\alpha_t+\sum_{\tau=0}^{t-1}\beta_\tau\right) - (1-\delta_{\tau,0})\,c\left(1-\sum_{\tau=0}^{t}\alpha_\tau-\sum_{\tau=0}^{t-1}\beta_\tau\right)G_1\left(\sum_{\tau=0}^{t-2}\beta_\tau\right), \tag{20}$$

which eases later calculations. Note that the relative size of the GCC for ordinary percolation can be recovered by substituting $t=0$ into the above equation, yielding

$$n_{\mathrm{GCC}}(0) = 1 - G_0\left(\alpha_0\right). \tag{21}$$

F. The residual giant biconnected component

If we keep performing GAPR in a network until there is no AP left, depending on the initial structure of the network, it could be totally destructed or left with an RGB. The relative size of the RGB can be calculated as

$$n_{\mathrm{RGB}}(c) = \sum_{k=2}^{\infty}P(k)\sum_{s=2}^{k}\binom{k}{s}\left(1-\sum_{\tau=0}^{T}\alpha_\tau-\sum_{\tau=0}^{T}\beta_\tau\right)\sum_{\tau=0}^{T}\beta_\tau, \tag{22}$$

which is based on the following observations:

- For any node in the RGB, its neighbors can not be α_t-nodes at any t. Otherwise, this node is removable as an AP.

- Since the RGB is biconnected, each of its nodes must be connected to at least two other RGB nodes. The probability that an end node of a randomly chosen link belongs to the RGB is $1 - \sum_{\tau=0}^{T} \alpha_\tau - \sum_{\tau=0}^{T} \beta_\tau$.

The diagrammatic representation of Eq. 22 is shown in Supplementary Fig. 12. After some algebra, Eq. 22 can be rewritten in terms of generating functions as:

$$n_{\mathrm{RGB}}(c) = G_0\left(1 - \sum_{\tau=0}^{T} \alpha_\tau\right) - G_0\left(\sum_{\tau=0}^{T} \beta_\tau\right) - c\left(1 - \sum_{\tau=0}^{T} \alpha_\tau - \sum_{\tau=0}^{T} \beta_\tau\right) G_1\left(\sum_{\tau=0}^{T} \beta_\tau\right). \quad (23)$$

Note that the RGB is nothing but the GCC at the last time step T, we can also obtain the above equation by substituting $t = T$ and also $\alpha_T = 0$, $\beta_T = 0$, $\beta_{T-1} = 0$ into the Eq. 20 of $n_{\mathrm{GCC}}(t, c)$.

G. Canonical model networks

The developed theoretical framework works for complex networks with any prescribed degree distribution. In this work, we consider two canonical model networks: ER networks and SF networks, which have specific degree distributions.

1. *Erdős-Rényi network*

For ER random network, the degree distribution $P(k)$ in the thermodynamic limit follows the Poisson distribution, i.e.

$$P(k) = \frac{e^{-c} c^k}{k!}, \quad (24)$$

where $c = \sum_{k=0}^{\infty} k P(k)$ is the mean degree. The generating functions are given by

$$G_0(x) = G_1(x) = e^{c(x-1)}. \quad (25)$$

Substituting the above equation into Eqs. 8, 9, and 10, we obtain the dynamic equations of the GAPR process on ER network:

$$\alpha_0 = \exp\left[c\left(\alpha_0 - 1\right)\right] \quad (26)$$

$$\alpha_{t>0} = \exp\left[c\left(\alpha_t + \sum_{\tau=0}^{t-1} \beta_\tau - 1\right)\right] - \exp\left[c\left(\sum_{\tau=0}^{t-2} \beta_\tau - 1\right)\right] \quad (27)$$

$$\beta_t = \exp\left(-c\sum_{\tau=0}^{t-1}\alpha_\tau\right) - \exp\left(-c\sum_{\tau=0}^{t}\alpha_\tau\right) - \exp\left[c\left(\alpha_t + \sum_{\tau=0}^{t-1}\beta_\tau - 1\right)\right] + \exp\left[c\left(\sum_{\tau=0}^{t-1}\beta_\tau - 1\right)\right]. \tag{28}$$

Similarly, the fraction of APs is given by

$$n_{\mathrm{AP}}(t,c) = \exp\left(-c\sum_{\tau=0}^{t-1}\alpha_\tau\right) - \exp\left(-c\sum_{\tau=0}^{t}\alpha_\tau\right) - c\alpha_t \exp\left[c\left(\sum_{\tau=0}^{t-1}\beta_\tau - 1\right)\right]. \tag{29}$$

Hence the fraction of APs in the original network at $t = 0$ is given by

$$n_{\mathrm{AP}}(c) = n_{\mathrm{AP}}(0,c) = 1 - \exp\left(-c\alpha_0\right) - c\alpha_0 \exp\left(-c\right). \tag{30}$$

The relative size of the GCC at the t-th time step is given by

$$\begin{aligned} n_{\mathrm{GCC}}(t) =& \exp\left(-c\sum_{\tau=0}^{t-1}\alpha_\tau\right) - \exp\left[c\left(\alpha_t + \sum_{\tau=0}^{t-1}\beta_\tau - 1\right)\right] \\ & - \left(1-\delta_{\tau,0}\right) c \left(1 - \sum_{\tau=0}^{t}\alpha_\tau - \sum_{\tau=0}^{t-1}\beta_\tau\right) \exp\left[c\left(\sum_{\tau=0}^{t-2}\beta_\tau - 1\right)\right]. \end{aligned} \tag{31}$$

The relative size of the RGB is

$$n_{\mathrm{RGB}}(c) = \exp\left(-c\sum_{\tau=0}^{T}\alpha_\tau\right) - \exp\left[c\left(\sum_{\tau=0}^{T}\beta_\tau - 1\right)\right] - c\left(1 - \sum_{\tau=0}^{T}\alpha_\tau - \sum_{\tau=0}^{T}\beta_\tau\right)\exp\left[c\left(\sum_{\tau=0}^{T}\beta_\tau - 1\right)\right]. \tag{32}$$

2. Scale-free network

We use the static model to generate asymptotically SF networks with tunable network size N, mean degree c, and degree distribution exponent $\lambda > 2$ [54].

This model can be described as follows:

Step-1: We start with N isolated nodes, which are labeled from 1 to N. Each node is assigned a weight $p_i \sim i^{-a}$, where $a = \frac{1}{\lambda - 1}$.

Step-2: We independently pick up two nodes according to their assigned weights, and add a link between these two nodes if they have not been connected before. Self-links and double-links are forbidden.

Step-3: Repeat step-2 until $M = cN/2$ links have been added into the network.

In the thermodynamic limit, the degree distribution of the static model can be analytically derived [55, 56]:

$$P(k) = \frac{[c(1-a)]^k}{ak!} \int_1^\infty dx \exp[-c(1-a)x] x^{k-1-1/a}. \tag{33}$$

The generating function of the degree distribution $P(k)$ is given by

$$\begin{aligned} G_0(x) =& \sum_{k=0}^{\infty} \frac{[c(1-a)]^k}{ak!} \int_1^\infty dy \exp[-c(1-a)y] y^{k-1-1/a} x^k \\ =& \frac{1}{a} \int_1^\infty dy \exp[-c(1-a)y] y^{-1-1/a} \sum_{k=0}^{\infty} \frac{[c(1-a)xy]^k}{k!} \\ =& \frac{1}{a} \int_1^\infty dy \exp[-c(1-a)(1-x)y] y^{-1-1/a}, \end{aligned} \tag{34}$$

where $e^{c(1-a)xy} = \sum_{k=0}^{\infty} \frac{[c(1-a)xy]^k}{k!}$ is used in the last step. Similarly, the generation function of the excess degree distribution $Q(k)$ is derived as

$$G_1(x) = \frac{G_0'(x)}{G_0'(1)} = \frac{1-a}{a} \int_1^\infty dy \exp[-c(1-a)(1-x)y] y^{-1/a}. \tag{35}$$

By defining

$$E_n(x) = \int_1^\infty e^{-xy} y^{-n} dy, \tag{36}$$

$G_0(x)$ and $G_1(x)$ can be rewritten as

$$G_0(x) = \frac{1}{a} E_{1+\frac{1}{a}}[c(1-a)(1-x)] \tag{37}$$

$$G_1(x) = \frac{1-a}{a} E_{\frac{1}{a}}[c(1-a)(1-x)] \tag{38}$$

where the fact $\frac{\partial E_n(x)}{\partial x} = -E_{n-1}(x)$ has been used.

Substituting the above equations into Eqs. 8, 9, and 10, we obtain the dynamic equations of the GAPR process for the static model:

$$\alpha_0 = \frac{1-a}{a} E_{\frac{1}{a}} \left[c\,(1-a)\,(1-\alpha_0) \right] \tag{39}$$

$$\alpha_{t>0} = \frac{1-a}{a} \left\{ E_{\frac{1}{a}} \left[c\,(1-a) \left(1 - \alpha_t - \sum_{\tau=0}^{t-1} \beta_\tau \right) \right] - E_{\frac{1}{a}} \left[c\,(1-a) \left(1 - \sum_{\tau=0}^{t-2} \beta_\tau \right) \right] \right\} \tag{40}$$

$$\begin{aligned} \beta_t =& \frac{1-a}{a} \Bigg\{ E_{\frac{1}{a}} \left[c\,(1-a) \sum_{\tau=0}^{t-1} \alpha_\tau \right] - E_{\frac{1}{a}} \left[c\,(1-a) \sum_{\tau=0}^{t} \alpha_\tau \right] \\ & - E_{\frac{1}{a}} \left[c\,(1-a) \left(1 - \alpha_t - \sum_{\tau=0}^{t-1} \beta_\tau \right) \right] - E_{\frac{1}{a}} \left[c\,(1-a) \left(1 - \sum_{\tau=0}^{t-1} \beta_\tau \right) \right] \Bigg\}. \end{aligned} \tag{41}$$

Similarly, the fraction of APs is given by

$$\begin{aligned} n_{\mathrm{AP}}(t,c) =& \frac{1}{a}\left\{E_{1+\frac{1}{a}}\left[c\,(1-a)\sum_{\tau=0}^{t-1}\alpha_\tau\right] - E_{1+\frac{1}{a}}\left[c\,(1-a)\sum_{\tau=0}^{t}\alpha_\tau\right]\right\} \\ & -\frac{c\alpha_t(1-a)}{a}E_{\frac{1}{a}}\left[c\,(1-a)\left(1-\sum_{\tau=0}^{t-1}\beta_\tau\right)\right]. \end{aligned} \tag{42}$$

Hence the fraction of APs in the original network at $t=0$ is

$$n_{\mathrm{AP}}(c) = n_{\mathrm{AP}}(0,c) = 1 - \frac{1}{a}E_{1+\frac{1}{a}}[c(1-a)\alpha_0] - \frac{c\alpha_0(1-a)}{a}E_{\frac{1}{a}}[c(1-a)]. \tag{43}$$

The relative size of the GCC at the t-th time step is given by

$$\begin{aligned} n_{\mathrm{GCC}}(t) =& \frac{1}{a}\left\{E_{1+\frac{1}{a}}\left[c\,(1-a)\sum_{\tau=0}^{t-1}\alpha_\tau\right] - E_{1+\frac{1}{a}}\left[c\,(1-a)\left(1-\alpha_t-\sum_{\tau=0}^{t-1}\beta_\tau\right)\right]\right\} \\ & -(1-\delta_{\tau,0})\,\frac{c(1-a)}{a}\left(1-\sum_{\tau=0}^{t}\alpha_\tau-\sum_{\tau=0}^{t-1}\beta_\tau\right)E_{\frac{1}{a}}\left[c\,(1-a)\left(1-\sum_{\tau=0}^{t-2}\beta_\tau\right)\right]. \end{aligned} \tag{44}$$

The relative size of the RGB is

$$\begin{aligned} n_{\mathrm{RGB}}(c) =& \frac{1}{a}\left\{E_{1+\frac{1}{a}}\left[c\,(1-a)\sum_{\tau=0}^{T}\alpha_\tau\right] - E_{1+\frac{1}{a}}\left[c\,(1-a)\left(1-\sum_{\tau=0}^{T}\beta_\tau\right)\right]\right\} \\ & -\frac{c(1-a)}{a}\left(1-\sum_{\tau=0}^{T}\alpha_\tau-\sum_{\tau=0}^{T}\beta_\tau\right)E_{\frac{1}{a}}\left[c\,(1-a)\left(1-\sum_{\tau=0}^{T}\beta_\tau\right)\right]. \end{aligned} \tag{45}$$

Supplementary Note 5 - Behavior of $c_{\mathbf{AP}}$ for SF Networks

According to the process of adding links in the static model, we can qualitatively explain the behavior of $n_{\rm AP}(c)$ and $c_{\rm AP}$ for SF networks with degree exponent λ generated by the static model. Compared with ER networks where links are added uniformly, in SF networks generated by the static model the link density between hubs is much higher. This important feature directly leads to three properties: (1) As we add more links, those hubs are quickly connected to each other and form a giant component, which results in smaller percolation mean degree $c_{\rm P}(\lambda)$ than that of ER networks. (2) Since a large fraction of nodes in the static model have small weights (and degrees), the link density between them is low. For $c > c_{\rm P}(\lambda)$, except the GCC, the rest of the network is so fragmented that the sizes of the FCCs are very small. Note that all the FCCs are trees in thermodynamics limit, because the probability of adding a link between two nodes with small weights in the same FCC to form a loop is nearly zero. (3) Since the FCCs are very small, in the process of adding links the GCC grows very slowly. It indicates that compared with ER networks, we have to add more links for the GCC to reach a critical size so that the contributions of type-I links (links inside the GCC) overwhelms that of type-II links (links that connect the GCC with an FCC or connect two FCCs), which results in a larger $c_{\rm AP}(\lambda)$. The above properties explain why the distance between $c_{\rm P}(\lambda)$ and $c_{\rm AP}(\lambda)$ is larger than that for ER network, as well as the observation that $c_{\rm AP}(\lambda) > c_{\rm P}(\lambda)$ is more prominent for smaller λ.

Supplementary Note 6 - Numerical Simulations

We have performed extensive numerical simulations to confirm our analytical results.

Supplementary Figure 4 shows the simulation results of the fraction of APs, $n_{\rm AP}(c,t)$, as a function of mean degree c at different time steps for ER networks and SF networks (constructed from the ER model and the static model) with different degree exponents λ. For comparison, analytical results for infinitely large networks are also shown (in lines).

The comparison between simulation results and analytical predictions of the relative size of the GCC, $n_{\rm GCC}(c,t)$, is shown in Supplementary Fig. 5. Note that, in Supplementary Figs. 4 and 5, the deviation of the simulation results from the theoretical prediction for SF networks with exponent $\lambda = 2.5$ owes to degree correlations in the constructed scale-free networks, which become prominent as $\lambda \to 2$ [57].

In Supplementary Fig. 6, we show the simulation results of the distribution of the relative size of the RGB, $n_{\rm RGB}$, at criticality for both ER network and SF networks with different degree exponents. The bimodal distribution is another evidence that $n_{\rm RGB}$ undergoes a discontinuous jump from zero to a large finite value at the critical point [49].

Supplementary Note 7 - Network Datasets

All the real-world networks analyzed in this work are listed and briefly described in the Supplementary Tables 1-14. For each network, we show its name and reference; number of nodes (N) and edges (L); and a brief description (unless it is clear from the network type).

SUPPLEMENTARY REFERENCES

[1] Colizza, V., Pastor-Satorras, R. & Vespignani, A. Reaction–diffusion processes and metapopulation models in heterogeneous networks. *Nature Physics* **3**, 276–282 (2007).

[2] Opsahl, T. Why anchorage is not (that) important: Binary ties and sample selection. Available at http://toreopsahl.com/2011/08/12/why-anchorage-is-not-that-important-binary-ties-and-sample-selection/ (2011).

[3] Leskovec, J. & Krevl, A. SNAP Datasets: Stanford Large Network Dataset Collection. Available at https://snap.stanford.edu/data/ (2014).

[4] Bianconi, G., Gulbahce, N. & Motter, A. E. Local structure of directed networks. *Physical Review Letters* **100**, 118701 (2008).

[5] Watts, D. J. & Strogatz, S. H. Collective dynamics of 'small-world' networks. *Nature* **393**, 440–442 (1998).

[6] Milo, R. *et al.* Network motifs: simple building blocks of complex networks. *Science* **298**, 824–827 (2002).

[7] Albert, R., Jeong, H. & Barabási, A.-L. Internet: Diameter of the world-wide web. *Nature* **401**, 130–131 (1999).

[8] Ulanowicz, R., Bondavalli, C. & Egnotovich, M. Network analysis of trophic dynamics in south florida ecosystem, fy 97: The florida bay ecosystem. *Annual Report to the United States Geological Service Biological Resources Division Ref. No.[UMCES] CBL* 98–123 (1998).

[9] Baird, D. & Ulanowicz, R. E. The seasonal dynamics of the Chesapeake Bay ecosystem. *Ecological Monographs* **59**, 329–364 (1989).

[10] Hagy, J. D. *Eutrophication, hypoxia and trophic transfer efficiency in Chesapeake Bay* (PhD Dissertation, University of Maryland at College Park, 2002).

[11] Ulanowicz, R. E. *Growth and development: ecosystems phenomenology* (Springer Science & Business Media, 2012).

[12] Pajek Food Web datasets. http://vlado.fmf.uni-lj.si/pub/networks/data/bio/foodweb/foodweb.htm.

[13] Ulanowicz, R., Bondavalli, C., Heymans, J. & Egnotovich, M. Network Analysis of Trophic Dynamics in South Florida Ecosystem, FY 99: The Graminoid Ecosystem. *Annual Report to the United States Geological Service Biological Resources Division Ref. No.[UMCES] CBL 00-0176, Chesapeake Biological Laboratory, University of Maryland* (2000).

[14] Dunne, J. A., Williams, R. J. & Martinez, N. D. Food-web structure and network theory: the role of connectance and size. *Proceedings of the National Academy of Sciences* **99**, 12917–12922 (2002).

[15] Martinez, N. D. Artifacts or attributes? Effects of resolution on the Little Rock Lake food web. *Ecological Monographs* **61**, 367–392 (1991).

[16] Patrício, J. M. *How well do ecological indicators assess environmental status?: case studies in coastal and estuarine ecosystems* (PhD thesis, University of Coimbra, Coimbra, Portugal, 2005).

[17] Almunia, J., Basterretxea, G., Arıstegui, J. & Ulanowicz, R. Benthic-pelagic switching in a coastal subtropical lagoon. *Estuarine, Coastal and Shelf Science* **49**, 363–384 (1999).

[18] Christian, R. R. & Luczkovich, J. J. Organizing and understanding a winter's seagrass foodweb network through effective trophic levels. *Ecological Modelling* **117**, 99–124 (1999).

[19] Memmott, J., Martinez, N. & Cohen, J. Predators, parasitoids and pathogens: species richness, trophic generality and body sizes in a natural food web. *Journal of Animal Ecology* **69**, 1–15 (2000).

[20] Baird, D., Luczkovich, J. & Christian, R. R. Assessment of spatial and temporal variability in ecosystem attributes of the St Marks National Wildlife Refuge, Apalachee Bay, Florida. *Estuarine, Coastal and Shelf Science* **47**, 329–349 (1998).

[21] Goldwasser, L. & Roughgarden, J. Construction and analysis of a large Caribbean food web. *Ecology* 1216–1233 (1993).

[22] Balaji, S., Babu, M. M., Iyer, L. M., Luscombe, N. M. & Aravind, L. Comprehensive analysis of combinatorial regulation using the transcriptional regulatory network of yeast. *Journal of Molecular Biology* **360**, 213–227 (2006).

[23] Gama-Castro, S. *et al.* RegulonDB (version 6.0): gene regulation model of Escherichia coli K-12 beyond transcription, active (experimental) annotated promoters and Textpresso navigation. *Nucleic Acids Research* **36**, D120–D124 (2008).

[24] Stark, C. *et al.* BioGRID: a general repository for interaction datasets. *Nucleic Acids Research* **34**, D535–D539 (2006).

[25] Song, C., Qu, Z., Blumm, N. & Barabási, A.-L. Limits of predictability in human mobility. *Science* **327**, 1018–1021 (2010).

[26] Eckmann, J.-P., Moses, E. & Sergi, D. Entropy of dialogues creates coherent structures in e-mail traffic. *Proceedings of the National Academy of Sciences* **101**, 14333–14337 (2004).

[27] Opsahl, T. & Panzarasa, P. Clustering in weighted networks. *Social Networks* **31**, 155–163 (2009).

[28] Richardson, M., Agrawal, R. & Domingos, P. Trust management for the semantic web. In *International semantic Web conference*, 351–368 (Springer, 2003).

[29] Van Duijn, M. A., Zeggelink, E. P., Huisman, M., Stokman, F. N. & Wasseur, F. W. Evolution of sociology freshmen into a friendship network. *Journal of Mathematical Sociology* **27**, 153–191 (2003).

[30] Milo, R. *et al.* Superfamilies of evolved and designed networks. *Science* **303**, 1538–1542 (2004).

[31] Freeman, S. C. & Freeman, L. C. *The networkers network: A study of the impact of a new communications medium on sociometric structure* (School of Social Sciences University of Calif., 1979).

[32] Cross, R. L. & Parker, A. *The hidden power of social networks: Understanding how work really gets done in organizations* (Harvard Business Review Press, 2004).

[33] Tarjan, R. E. & Vishkin, U. An efficient parallel biconnectivity algorithm. *SIAM Journal on Computing* **14**, 862–874 (1985).

[34] Albert, R., Jeong, H. & Barabási, A.-L. Error and attack tolerance of complex networks. *Nature* **406**, 378–382 (2000).

[35] Cohen, R., Erez, K., Ben-Avraham, D. & Havlin, S. Resilience of the Internet to random breakdowns. *Physical Review Letters* **85**, 4626 (2000).

[36] Morone, F. & Makse, H. A. Influence maximization in complex networks through optimal percolation. *Nature* **524**, 65–68 (2015).

[37] Borgatti, S. P. & Everett, M. G. Models of core/periphery structures. *Social Networks* **21**, 375–395 (2000).

[38] Rombach, M. P., Porter, M. A., Fowler, J. H. & Mucha, P. J. Core-periphery structure in networks. *SIAM Journal on Applied Mathematics* **74**, 167–190 (2014).

[39] Zhang, X., Martin, T. & Newman, M. E. Identification of core-periphery structure in networks. *Physical Review E* **91**, 032803 (2015).

[40] Verma, T., Russmann, F., Araújo, N., Nagler, J. & Herrmann, H. Emergence of core-peripheries in networks. *Nature Communications* **7**, 10441 (2016).

[41] Behzad, M. & Chartrand, G. *Introduction to the Theory of Graphs* (Allyn and Bacon, 1972).

[42] Harary, F. *Graph Theory* (Addison-Wesley, Reading, MA, 1969).

[43] Dorogovtsev, S. N., Goltsev, A. V. & Mendes, J. F. F. K-core organization of complex networks. *Physical Review Letters* **96**, 040601 (2006).

[44] Kitsak, M. *et al.* Identification of influential spreaders in complex networks. *Nature Physics* **6**, 888–893 (2010).

[45] Zhang, Y. & Parthasarathy, S. Extracting analyzing and visualizing triangle k-core motifs within networks. In *2012 IEEE 28th International Conference on Data Engineering*, 1049–1060 (IEEE, 2012).

[46] Verma, T., Araújo, N. A. & Herrmann, H. J. Revealing the structure of the world airline network. *Scientific Reports* **4** (2014).

[47] Callaway, D. S., Newman, M. E. J., Strogatz, S. H. & Watts, D. J. Network robustness and fragility: Percolation on random graphs. *Physical Review Letters* **85**, 5468 (2000).

[48] Newman, M. E. J., Strogatz, S. H. & Watts, D. J. Random graphs with arbitrary degree distributions and their applications. *Physical Review E* **64**, 026118 (2001).

[49] Dorogovtsev, S. N., Goltsev, A. V. & Mendes, J. F. Critical phenomena in complex networks. *Reviews of Modern Physics* **80**, 1275 (2008).

[50] Mezard, M. & Montanari, A. *Information, physics, and computation* (Oxford University Press, 2009).

[51] Melnik, S., Hackett, A., Porter, M. a., Mucha, P. J. & Gleeson, J. P. The unreasonable effectiveness of tree-based theory for networks with clustering. *Physical Review E* **83**, 36112 (2011).

[52] Albert, R. & Barabási, A.-L. Statistical mechanics of complex networks. *Reviews of Modern Physics* **74**, 47 (2002).

[53] Newman, M. E. J. The structure and function of complex networks. *SIAM Review* **45**, 167–256 (2003).

[54] Goh, K.-I., Kahng, B. & Kim, D. Universal behavior of load distribution in scale-free networks. *Physical Review Letters* **87**, 278701 (2001).

[55] Catanzaro, M. & Pastor-Satorras, R. Analytic solution of a static scale-free network model. *The European Physical Journal B-Condensed Matter and Complex Systems* **44**, 241–248 (2005).

[56] Lee, J.-S., Goh, K.-I., Kahng, B. & Kim, D. Intrinsic degree-correlations in the static model of scale-free networks. *The European Physical Journal B-Condensed Matter and Complex Systems* **49**, 231–238 (2006).

[57] Boguná, M., Pastor-Satorras, R. & Vespignani, A. Cut-offs and finite size effects in scale-free networks. *The European Physical Journal B-Condensed Matter and Complex Systems* **38**, 205–209 (2004).
